# Delta and gamma oscillations in operculo-insular cortex underlie innocuous cold thermosensation

**DOI:** 10.1152/jn.00843.2016

**Published:** 2017-03-01

**Authors:** Francesca Fardo, Mikkel C. Vinding, Micah Allen, Troels Staehelin Jensen, Nanna Brix Finnerup

**Affiliations:** ^1^Danish Pain Centre, Department of Clinical Medicine, Aarhus University, Aarhus, Denmark;; ^2^Interacting Minds Centre, Aarhus University, Aarhus, Denmark;; ^3^Center of Functionally Integrative Neuroscience, Aarhus University, Aarhus, Denmark;; ^4^Swedish National Facility for Magnetoencephalography, Department of Clinical Neuroscience, Karolinska Institutet, Stockholm, Sweden;; ^5^Wellcome Trust Center for Neuroimaging, University College London, London, United Kingdom;; ^6^Institute of Cognitive Neuroscience, University College London, London, United Kingdom; and; ^7^Department of Neurology, Aarhus University Hospital, Aarhus, Denmark

**Keywords:** cold, cold-evoked responses, magnetoencephalography, EEG

## Abstract

Cold-sensitive and nociceptive neural pathways interact to shape the quality and intensity of thermal and pain perception. Yet the central processing of cold thermosensation in the human brain has not been extensively studied. Here, we used magnetoencephalography and EEG in healthy volunteers to investigate the time course (evoked fields and potentials) and oscillatory activity associated with the perception of cold temperature changes. Nonnoxious cold stimuli consisting of Δ3°C and Δ5°C decrements from an adapting temperature of 35°C were delivered on the dorsum of the left hand via a contact thermode. Cold-evoked fields peaked at around 240 and 500 ms, at peak latencies similar to the N1 and P2 cold-evoked potentials. Importantly, cold-related changes in oscillatory power indicated that innocuous thermosensation is mediated by oscillatory activity in the range of delta (1–4 Hz) and gamma (55–90 Hz) rhythms, originating in operculo-insular cortical regions. We suggest that delta rhythms coordinate functional integration between operculo-insular and frontoparietal regions, while gamma rhythms reflect local sensory processing in operculo-insular areas.

**NEW & NOTEWORTHY** Using magnetoencephalography, we identified spatiotemporal features of central cold processing, with respect to the time course, oscillatory profile, and neural generators of cold-evoked responses in healthy human volunteers. Cold thermosensation was associated with low- and high-frequency oscillatory rhythms, both originating in operculo-insular regions. These results support further investigations of central cold processing using magnetoencephalography or EEG and the clinical utility of cold-evoked potentials for neurophysiological assessment of cold-related small-fiber function and damage.

## Abstract (precis)

Using magnetoencephalography, we identified spatiotemporal features of central cold processing, with respect to the time course, oscillatory profile, and neural generators of cold-evoked responses in healthy human volunteers. Cold thermosensation was associated with low- and high-frequency oscillatory rhythms, both originating in operculo-insular regions. These results support further investigations of central cold processing using magnetoencephalography or EEG and the clinical utility of cold-evoked potentials for neurophysiological assessment of cold-related small-fiber function and damage.

intact thermosensation is critical to maintain optimal homeostatic function and prevent bodily damage. For example, the interaction of cold thermosensation and nociception in the central nervous system is important for determining the quality and intensity of perceived pain. Normally, innocuous cold inputs exert analgesic effects on nociception (e.g., cold-induced analgesia; Bini et al. 1984), but nonpainful cold can also elicit paradoxical heat and pain sensations, as, for example, in the thermal grill illusion (Thunberg 1896). On the basis of this interaction, disrupted processing of cold signals has been suggested to be a potential mechanism responsible for cold allodynia in central neuropathic pain (Craig 1998). A detailed understanding of cortical dynamics mediating cold processing will therefore establish a basis for identifying pathophysiological mechanisms associated with cold-related neuropathic symptoms. In the present study, we leverage the high spatial and temporal resolution afforded by magnetoencephalography to identify distinct features of cold-related activity in terms of time course (evoked fields and potentials), oscillations, and source localization of cold-related responses. This study thus provides insight into the latency, morphology, and localization of cold-related responses and oscillations, providing a useful basis for future studies comparing cold-related neural activity in patients with cold allodynia and healthy controls.

Innocuous cold inputs are transmitted in the central nervous system via spinothalamic fibers, composed of distinct small-diameter A-delta and C fiber afferents, which project to thalamic and operculo-insular cortical sites [for review, see Yin et al. (2015)]. In humans, the notion that cold-sensitive fibers project to operculo-insular regions is supported by converging evidence from intracranial stimulation (Ostrowsky et al. 2002) and recordings (Greenspan et al. 2008), as well as functional neuroimaging (Craig et al. 2000; Hua et al. 2005; Mazzola et al. 2012) and lesion studies (Greenspan et al. 1999). Beyond operculo-insular regions, cold thermosensation is also related to an extended network of brain areas including the midanterior insula, somatosensory, frontal, and parietal regions [Craig et al. 2000; Hua et al. 2005; see also Oshiro et al. (2007) and Oshiro et al. (2009)]. Although these studies provide a relatively clear picture of the neuroanatomy underlying thermosensation, little is known about the temporal and oscillatory features of cold-related neural responses.

A potential remedy to this deficit is to use EEG and magnetoencephalography (MEG) to identify the timing (cold-evoked potentials and fields) and pattern of synchronization and desynchronization of brain oscillations underlying cold sensory processing. EEG and MEG directly measure electrical and magnetic neural activity produced by apical dendrites and other membrane potentials (Baillet et al. 2001). These techniques provide insight into the coordinated behavior of populations of neurons underlying perception, cognition, behavior, and disruptions thereof (Başar and Güntekin 2008; Başar et al. 2000). On the basis of the physics of oscillatory phenomena, low- and high-frequency neural oscillations can be linked to neural mechanisms occurring at distinct temporal and spatial scales (Buzsáki and Draguhn 2004). Low-frequency brain oscillations (e.g., delta, theta, and alpha) mediate long-range communication at slow timescales across distant brain regions and are crucial for functional integration in large-scale brain networks. In contrast, high-frequency brain oscillations (e.g., gamma) are more transient and focal and thus important for local neuronal synchrony in cortical areas (Canolty and Knight 2010). Understanding these spatiotemporal and oscillatory aspects in the context of cold-related neural responses will therefore inform the neural mechanisms underlying cold thermosensation.

Recent reviews and experimental work suggest the possibility of recording cold-evoked potentials using a contact thermode designed to elicit heat-evoked potentials (Baumgärtner et al. 2012; Hüllemann et al. 2016; Maihöfner et al. 2002). Specifically, Hüllemann et al. (2016) have demonstrated that cold-evoked potentials reflect A-delta fiber integrity, loss of function, and functional recovery in healthy participants. Here, we used this stimulation apparatus in combination with MEG and EEG recordings to investigate evoked and oscillatory neural activity associated with central cold processing and their neural generators in source space. In the time domain, we expected to observe evoked fields in the time range of cold-related N1, N2, and P2 potentials, between 100 and 600 ms (Baumgärtner et al. 2012; Hüllemann et al. 2016; Maihöfner et al. 2002). In the time-frequency domain, we expected synchronization at low delta-to-theta and high gamma frequencies, reflecting global and local functional integration, respectively. We expected that low-frequency oscillations would originate in both cold sensory (opercular) and attention-related (frontoparietal) regions, reflecting long-range functional integration as driven by executive task demands. Furthermore, we anticipated that synchronization of high-frequency activity would emerge in opercular regions, reflecting local sensory computations underlying cold thermosensation.

## METHODS

### Participants

Six healthy volunteers [3 women; age 23.5 ± 1.23 (mean ± SD) yr; range 20–27 yr] were recruited from Aarhus University and the local community. All participants were right handed, with normal or corrected-to-normal vision. No participants reported a history of pain disorders or neurological or psychiatric illness. All participants received a reimbursement of 300 DKK (~44 USD) for participation and gave written informed consent before participation. The study was approved by the Ethical Committee of Central Region Denmark and conducted in accordance with the Declaration of Helsinki.

### Cooling Stimuli and Behavioral Task

**Fig. 1.**
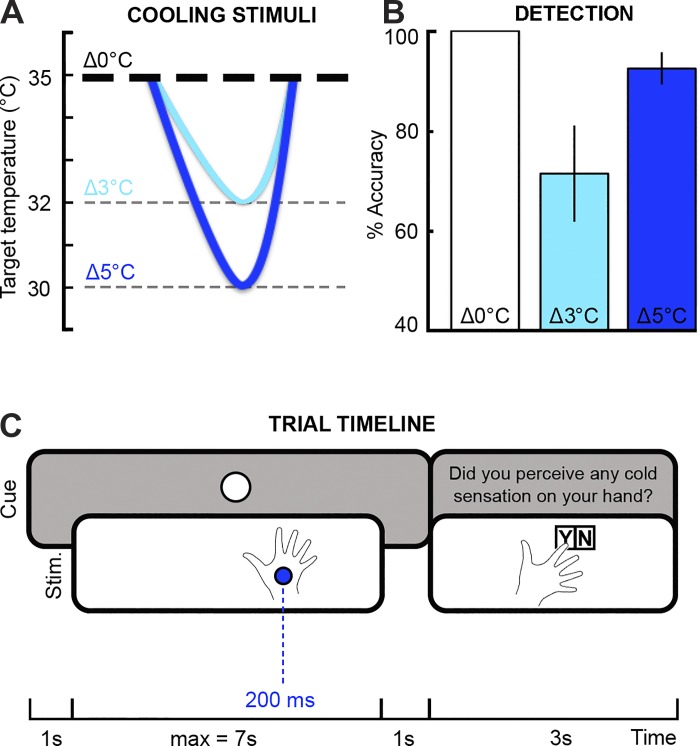
*A*: experimental stimuli consisted of decrements of 5°C (dark blue) and 3°C (light blue) from a baseline temperature of 35°C (bold dashed line). Eighty stimuli were delivered for each temperature change in a random order. The stimulus trials were pseudorandomly intermixed with 40 catch trials where no stimulus was delivered (i.e., temperature remained constant at 35°C). *B*: mean and SE of detection accuracy for catch (Δ0°C) and cold stimulus trials (Δ5 and Δ3°C). *C*: timeline of a single trial. Each trial started with the presentation of a visual cue (either a white or a black circle on a gray background) at the center of the screen. The cue duration was between 2 and 9 s. In the experimental trials, the cold stimuli (stim.) were presented at least 1 s after and 1 s before cue onset and offset, respectively. After the cue disappeared, participants were asked to report whether they felt any cold sensation with a yes/no (Y/N) answer (i.e., delayed-response task). Max, maximum.

Cold-evoked responses were elicited by mild cooling of the dorsum of the left hand using a Peltier-based contact thermode [Pathway model Contact Heat-Evoked Potential Stimulator (CHEPS); Medoc, Haifa, Israel]. The thermode consisted of a 9-cm^2^ surface and was adapted for use in a MEG or magnetic resonance (MR) scanner environment via a physical filter, to minimize the influence of stimulation-related electric and magnetic artifacts on the acquired MEG signal. Although CHEPS is optimally designed to deliver warm and heat stimuli, it can be programmed to generate steep cooling temperature ramps from a baseline temperature of 35°C to a target temperature of 30°C. To elicit synchronized neural responses, we used the maximal rate of 70°C/s with a return rate of 40°C/s, eliciting a thermal change of ~200 ms. Two sets of cooling stimuli (Fig. 1, *A* and *B*) were pseudorandomly delivered on a fixed position on the dorsum of the left hand: *1*) 80 stimuli corresponding to a decrement of 5°C from a baseline temperature of 35°C (target temperature = 30°C), and *2*) 80 stimuli corresponding to a decrement of 3°C from a baseline temperature of 35°C (target temperature = 32°C). The cooling stimuli were pseudorandomly interspersed with 40 catch trials where no stimulus was presented.

Participants were instructed to covertly attend to their left hand while a visual cue was presented at the center of a screen. The visual cue consisted of a white or black circle presented on a gray background for 2–9 s. In the experimental trials (80% of trials), a cooling stimulus was delivered between 1 and 8 s after cue onset and lasted for ~200 ms. In the catch trials (remaining 20% of trials), no cooling stimulus was delivered. After the cue disappeared, participants were asked to report whether they felt a cold sensation during the cue period (i.e., delayed-response task). Each yes/no response was indicated by pressing one of two buttons with either the right index or middle finger. Participants had 3 s maximum to provide an answer. The intercue interval was fixed at 3 s, while the jittered interstimulus interval was on average 9.56 s (minimum = 5; maximum = 19 s). The long interstimulus intervals were chosen to lessen possible habituation effects, while the detection task ensured that participants attended to each stimulus trial. The entire duration of the task was ~30–35 min. The PsychoPy software package v1.76.00 (Peirce 2007; Peirce 2009) was used to display visual inputs (i.e., written instructions and cue presentation), trigger the CHEPS stimulation, and collect behavioral responses (i.e., yes/no answer in the delayed-response task).

### Behavioral Analysis

To analyze response accuracy in the delayed detection task, we used a 2 × 2 contingency table, where rows represented the two levels of cooling intensity (Δ3 and Δ5°C) while columns depicted the two levels of responses (yes/detection vs. no/miss). We thus calculated whether the average number of detected cold stimuli varied depending on cooling intensity. The two-tailed *P* value was calculated with McNemar's test with the continuity correction. In addition, to investigate habituation of cooling perception, we fitted a binomial logistic regression model to each participant's single-trial accuracy data. Our regression models included three regressors of interest consisting of z-scored trial number, z-scored condition number, and their interaction (i.e., stimulus condition × trial time). We assessed whether detection accuracy decreased over time and whether habituation was significantly different across the two stimulus intensities. Statistical significance was calculated by using one-sample *t*-tests on the standardized logistic regression beta values.

### MEG/EEG Acquisition and Analysis

MEG data were acquired using an Elekta Neuromag TRIUX MEG system with 204 planar gradiometers and 102 magnetometers. EEG data were recorded simultaneously using an integrated 64-channel EEG system consisting of passive electrodes mounted on an elastic cap (Elekta Neuromag). Blinks and eye movements were monitored using vertical and horizontal bipolar surface electrodes. The data were digitized with a sample frequency of 1,000 Hz, with analog filtering of 0.1–330 Hz. A continuous measure of the head position with respect to the sensors was obtained using four head position indicator coils attached to the scalp. Furthermore, 3 fiducial markers (nasion and left and right preauricular points) and around 100 scalp points were digitized to define a common coordinate frame to superimpose functional MEG onto individual structural MRI data, recorded with a Siemens 3T MR scanner using a standard T1-weighted sequence.

We applied MaxFilter 2.2.15 software (Elekta Neuromag) on raw MEG data to *1*) remove externally generated noise using the temporal extension of the signal source separation (tSSS) algorithm (Taulu et al. 2005), *2*) detect bad channels automatically, *3*) correct for head movements within session, and *4*) correct for head positions across participants. To ensure optimal interference suppression of artifactual activity generated by the thermal stimulator, we set the tSSS processing buffer length to 18 s (i.e., each segment included on average 2 stimulus-related artifacts), and we lowered the correlation limit for the subspace intersection to 0.9 (i.e., more conservative than the default parameter of 0.98). Using MNE-Python 0.8.6 software (Gramfort et al. 2013), we then applied independent component analysis on raw MaxFiltered MEG to correct for blinks and vertical eye movements. Data were then further preprocessed and analyzed using the FieldTrip toolbox (Oostenveld et al. 2011) and Statistical Parametric Mapping 12 (SPM12, http://www.fil.ion.ucl.ac.uk/spm).

In FieldTrip, we analyzed MEG/EEG data in the time and time-frequency domains. In the time domain analysis, continuous MEG/EEG data were low-pass filtered at 20 Hz, epoched from −0.5 to 1 s, and baseline corrected using the average activity of the prestimulus interval. In the time-frequency analysis, continuous MEG data were epoched from −1 to 1.5 s and baseline corrected using the average activity of the entire stimulus interval. No filter was applied. In both analyses, trials containing artifacts (e.g., due to movement) were discarded by visual inspection (using ft_rejectvisual.m).

#### Time domain analysis.

**Fig. 2.**
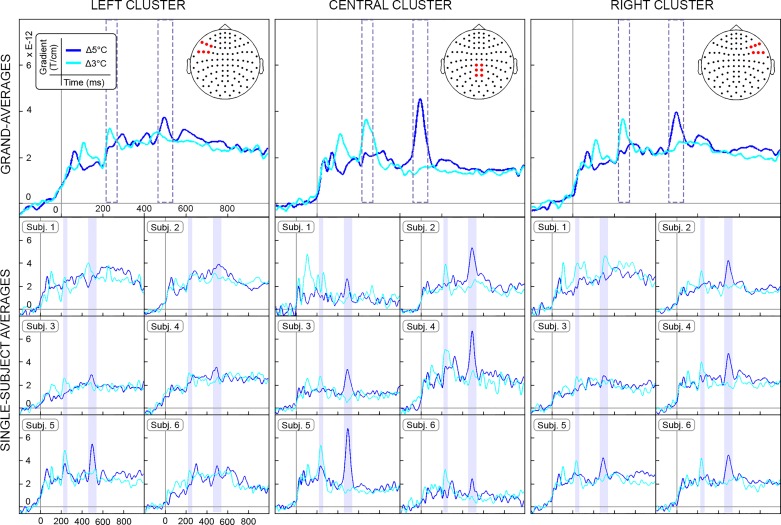
Grand average and single-subject average of the cold-evoked fields measured by combined planar gradiometers. The time courses are shown in one left (ipsilateral), one central, and one right (contralateral) cluster of sensors. Event-related fields are time locked to the onset of cold stimulation and displayed separately for the two levels of cooling intensity, i.e., Δ5°C (dark blue) and Δ3°C (light blue). Timescale (*x*-axis) is from −200 to 1,000 ms. Magnetic gradient (*y*-axis) is from −0.5 to 8 × *e^−^*^12^ T/cm. The time intervals of significant effects associated with the two cooling intensities (i.e., 213–267 and 456–544 ms) are displayed as blue rectangles. These intervals highlight highly consistent time courses and topographies of cold-evoked responses across all six participants in all groups of sensors.

To assess the overall effects of cold stimulation in the time domain, we averaged epochs time locked to the onset of perceived cold stimuli (pooled Δ5 and Δ3°C) and catch trials (i.e., nonstimulation). Furthermore, to assess differences in time-locked activity between the two cooling intensities, we separately averaged correctly detected Δ5 and Δ3°C cooling trials. Crucially, EEG data were rereferenced to the electrode Fz to analyze the latency of the cold-related N1 peak in temporal electrodes (T7 and T8) and to the mean of the left and right mastoids to analyze the latency of cold-related N2 and P2 peaks at the vertex (Cz). The analysis thus followed standard procedures for the assessment of nociceptive-specific evoked potentials (e.g., Treede et al. 2003). To establish whether EEG and MEG recordings reflected different neural activity, we compared the latency and morphology of EEG and MEG peaks. We selected MEG sensors overlapping the areas over the temporal cortex corresponding to T7 and T8 electrodes and overlapping the central-posterior region corresponding to Cz. The three clusters of MEG sensors are displayed in Fig. 2.

#### Time-frequency domain analysis.

We analyzed the time-frequency representations of cold-related neuromagnetic activity in the low- and high-frequency oscillatory range. Low-frequency oscillations between 1 and 30 Hz were calculated using a 500-ms Hanning window in steps of 50 ms in both baseline (−1 to 0 s) and stimulation (0–1 s) time windows. For normalization, we computed the absolute change in power with respect to the baseline period. The power spectrum was analyzed with respect to four frequency bands: delta (1–4 Hz), theta (4–8 Hz), alpha (8–13 Hz), and beta (13–30 Hz). Furthermore, high-frequency (i.e., gamma) oscillations between 30 and 100 Hz were calculated using a multitaper approach (Mitra and Pesaran 1999), by applying a fixed-length window of 400 ms in steps of 20 ms and a spectral smoothing of 5 Hz in both baseline (−1 to 0 s) and cold stimulation (0–1 s) time windows. For normalization, we computed the relative power change with respect to baseline. We analyzed the high-gamma frequency band between 55 and 90 Hz. The statistical analysis was identical as in the time domain and included two contrasts, i.e., cold stimulation (pooled Δ5 and Δ3°C) vs. nonstimulation trials and Δ5 vs. Δ3°C cooling stimuli. Both time and time-frequency statistical analyses at the scalp level were conducted on combined planar gradiometers. Evoked fields and power spectra of the two planar gradiometers were computed separately and then combined to provide estimates that are independent from the gradient orientation. The gradient topographies entail only positive values (i.e., absolute dipole moment, irrespective of its orientation) and provide an approximate estimate of the sources contributing to the neuromagnetic activity of interest, as the underlying sources are roughly underneath the peak of the magnetic gradient. We thus analyzed combined gradiometer data to maximize the sensitivity in detecting activity originating from similar sources across subjects, irrespective of dipole orientation, and to facilitate the interpretation of scalp topographies.

#### Source analysis.

Finally, we estimated the neural origin of the low-frequency and high-frequency oscillatory activity, in the poststimulation period between 0 and 1,000 ms, using an empirical Bayes beamformer method (Belardinelli et al. 2012), implemented in SPM12. After one unique source inversion, we analyzed three different sets of three-dimensional current density maps corresponding to the significant effects at the scalp level: *1*) 4-Hz delta-evoked activity between 100 and 350 ms, *2*) 4-Hz delta-evoked activity between 400 and 600 ms, and *3*) 60-Hz gamma-evoked activity between 340 and 600 ms. All images were smoothed with a low-pass kernel (8 mm × 8 mm × 8 ms; full width at half maximum). No statistic was conducted at the source level, as we based the analysis on statistically significant time-frequency effects at the scalp level. The contrasts of interest were inspected at an a priori threshold of *t* > 2. The source reconstruction analysis was performed on data from both magnetometers and planar gradiometers. Individual MRIs were available for all participants but one, for which a template brain was instead used. Anatomical labels for source locations were defined using cytoarchitectonic probabilistic maps from the SPM Anatomy Toolbox (Eickhoff et al. 2005).

#### Statistics.

In both time and time-frequency analysis, the effects of cold stimulation and the difference between the two cooling intensities were tested using a nonparametric permutation procedure in FieldTrip. The analysis was applied at each time point of the poststimulus interval of interest (i.e., 100–600 ms). This time window was consistent with expected cold-related activity between 100 and 600 ms following stimulation on the hand dorsum (Baumgärtner et al. 2012; Hüllemann et al. 2016; Maihöfner et al. 2002). In the time-frequency analysis, we analyzed separately each oscillatory band of interest (i.e., delta, 1–4 Hz; theta, 4–8 Hz; alpha, 8–13 Hz; beta, 13–30 Hz; high-gamma, 55–90 Hz). We controlled for multiple comparisons using a clustering algorithm (threshold of *P* = 0.05) repeated over 1,000 permutations.

## RESULTS

### Behavioral Results

Participants correctly identified all catch trials (Δ0°C) as noncooling stimuli and detected 71.5 (±9.6%) and 92.5% (±3.2%) of Δ3 and Δ5°C cooling changes, respectively (Fig. 1*C*). More specifically, participants identified between 40 and 100% of Δ3°C cooling stimuli and between 78.8 and 100% of Δ5°C cooling stimuli. The McNemar's test revealed that participants were on average more accurate in detecting Δ5°C compared with Δ3°C cooling stimuli [χ^2^(1) = 37.52, *P* < 0.0001]. Furthermore, habituation to cooling stimuli was significant over time [beta = −0.47 ± 0.41 (mean ± SD); *P* < 0.05]. However, the logistic regression on the temperature by time interaction was not significant (beta = 0.30 ± 0.24; *P* = 0.11), suggesting that habituation was not statistically different between the two levels of cooling intensity.

### MEG and EEG Results

#### Effects of cold stimulation in the time domain: cold-evoked fields and potentials.

**Fig. 3.**
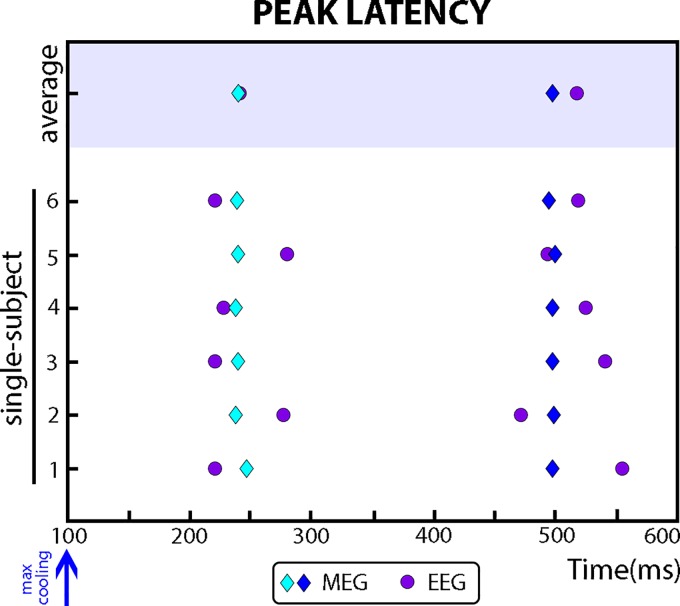
Grand average and single-subject average of cold-evoked responses' [magnetoencephalography (MEG) and EEG] latencies. In both EEG and MEG modalities, we observed two peaks at around 240 and 500 ms. The latency of the first was analyzed in right (contralateral) temporal channels (EEG-T8 and MEG-right cluster), whereas the latency of the second peak was analyzed in central channels (EEG-Cz and MEG-central cluster). We recorded similar EEG amplitudes elicited by Δ5 and Δ3°C cooling stimuli (purple circles). However, MEG signals differed depending on cooling intensity. Δ3°C stimuli elicited a greater peak at ~240 ms (light blue diamonds), while Δ5°C elicited a greater peak at ~500 ms (dark blue diamonds). Irrespective of the amplitude difference, EEG and MEG peak latencies were highly similar. We relate these results to the distinct spatial sensitivity offered by the two techniques.

The cluster-based permutation test revealed significant increments in amplitude (EEG) and field strength (MEG) by cold stimuli compared with nonstimulation trials. In EEG, cold-evoked potentials differed from nonstimulation trials between 342 and 600 ms (*P* < 0.005). This difference consisted of a large positive (cold-evoked P2) amplitude with maximal activity at the vertex. Interestingly, we did not observe consistent cold-evoked N2 potentials across all participants. However, we identified a clear cold-evoked N1 peak in temporal electrodes in Fz-referenced data. No significant differences were observed between the two cooling intensities at any time point. Thus both Δ5 and Δ3°C cooling stimuli elicited cold-evoked activity at similar latencies and amplitude. The latencies of the cold-evoked N1 potentials (pooled Δ5 and Δ3°C) were 241 ms (±5 ms) in the contralateral electrode (T8-Fz reference) and 244 ms (±5 ms) in the ipsilateral electrode (T7-Fz reference). Furthermore, the latency of the P2 cold-evoked potentials was 516 ms (±5 ms) at the vertex (Cz-mastoids reference; Fig. 3).

In MEG, cold-evoked fields differed from nonstimulation trials in two time windows: 100–304 ms (*P* < 0.01) and 464–565 ms (*P* < 0.05). The analysis also showed significant differences between the time courses of Δ5 and Δ3°C cooling. Increased field strength within the earlier time window was driven by Δ3°C cooling. The least cold stimuli elicited increased evoked activity between 213 and 267 ms (negative cluster, *P* < 0.01), over central-parietal and right frontotemporal sensors (Fig. 2). Furthermore, increased field strength within the later time window was driven by Δ5°C cooling. The coldest stimuli elicited increased evoked activity between 456 and 544 ms (positive cluster, *P* < 0.05). This latter difference was also more pronounced over central-parietal and right frontotemporal sensors (Fig. 2). In both cooling conditions, the locations of the global maxima were consistent with bilateral frontotemporal and parietal sources.

The latencies of cold-evoked peaks measured by EEG and MEG were striking similar (Fig. 3), suggesting that the contribution of radial sources usually undetected by MEG was negligible. Interestingly, MEG revealed different time courses for the two levels of cooling intensities, which were not identified by EEG. However, the neuromagnetic peak elicited by Δ3°C cooling at 240 ms (±5 ms) was consistent with the latency of the cold-evoked N1 potentials, while the neuromagnetic peak elicited by Δ5°C cooling at 497 ms (±2 ms) was consistent with the latency of the cold-evoked P2 potentials (Fig. 3). The consistent latencies suggest that MEG and EEG are measuring mostly similar neural activity, while the differences in amplitude are likely related to differences in spatial sensitivity offered by the two techniques (Hämäläinen et al. 1993).

#### Effects of cold stimulation in the time-frequency domain: cold-evoked oscillations.

**Fig. 4.**
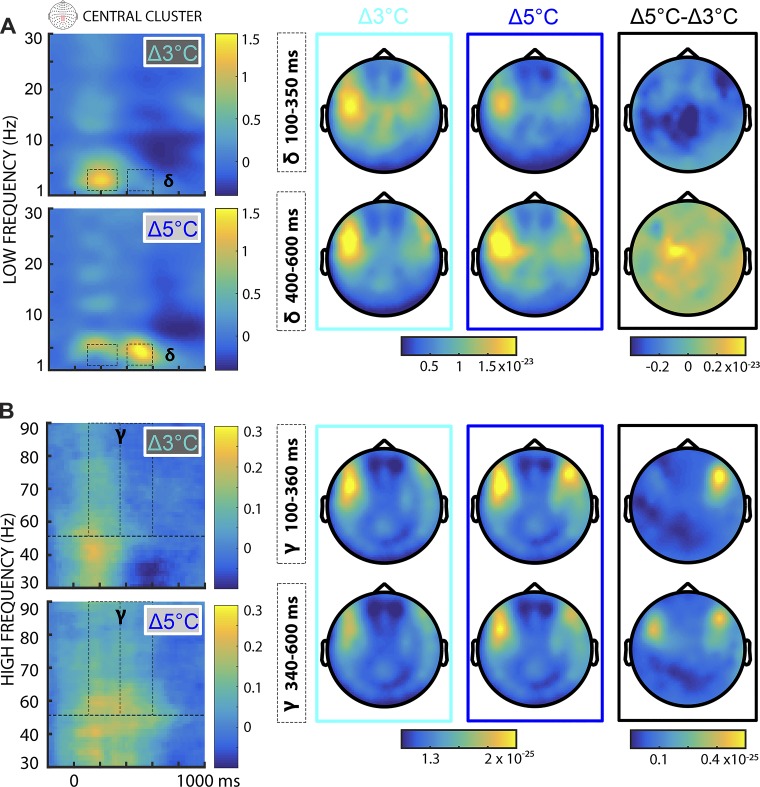
Topographic representation of time-frequency representations (TFRs). Average delta and gamma TFRs are depicted separately for Δ5°C, Δ3°C, and the difference between the two cooling temperatures (Δ5°C − Δ3°C). *A*: in the low-frequency range (i.e., delta, 1–4 Hz), we found significant differences between the two cooling intensities between 100 and 350 ms and between 400 and 600 ms. The difference was most pronounced over central and right temporal sensors. *B*: in the high-frequency range (i.e., gamma, 55–90 Hz), we observed significant effects only in a late time window, between 340 and 600 ms. The horizontal dashed line at 50 Hz indicates missing data (45–55 Hz) due to the application of a notch filter, during MaxFiltering.

With respect to nonstimulation trials, cold stimulation increased neuronal synchronization in the delta (1–4 Hz), theta (4–8 Hz), and gamma (55–90 Hz) frequency ranges, while they enhanced desynchronization in the alpha-to-beta (8–30 Hz) frequency ranges throughout the time window between 100 and 600 ms (all *P* < 0.05). Figure 4 shows the topographies of oscillatory changes separately for the two levels of cold stimulation in a central cluster of sensors. Furthermore, we found that delta band power was increased by both cold temperatures, but at different time points. Δ3°C cold stimuli evoked increased delta band oscillatory activity between 100 and 350 ms (negative cluster, *P* < 0.001). Conversely, Δ5°C cold stimuli evoked increased delta band oscillatory activity between 400 and 600 ms (positive cluster, *P* = 0.015). In both time windows, these time-frequency effects were observed in central-parietal and bilateral frontotemporal sensors. Crucially, the delta band enhancement effects were maximal in the same time windows and over the same sensors as in the time domain analysis. This pattern of results suggests that changes in delta band oscillations are a key component contributing to the timing and amplitude of cold-evoked responses.

Compared with nonstimulation trials, cold stimulation was also associated with increased theta synchronization and alpha-to-beta desynchronization (all *P* < 0.05). In particular, theta power was notably increased by Δ5 vs. Δ3°C cooling between 400 and 600 ms (positive cluster, *P* < 0.001), while no negative cluster was found. The strongest differences were found in central-parietal and frontotemporal regions. In addition, alpha and beta band power decrements were modulated by cooling intensity between 350 and 600 ms (Δ5 vs. Δ3°C, positive cluster, *P* < 0.001), with the greatest differential activity in central-parietal sensors.

Finally, cold stimuli elicited increased synchronization at higher frequencies, in the gamma band range. Gamma power was also modulated by cooling intensity, with the stronger cold stimulation leading to greater increments in gamma power between 340 and 600 ms (Δ5 vs. Δ3°C, positive cluster, *P* < 0.001). The topographies of the gamma responses were in bilateral frontotemporal regions, over similar sensors associated with oscillatory changes in delta-to-theta low-frequency rhythms. Overall, these results indicate that low- and high-frequency oscillatory rhythms, spanning from delta to gamma changes in power, are functionally linked to the processing of cold intensity. However, given the distinct intrinsic spatiotemporal properties of low- and high-frequency oscillations, lower rhythms might be involved in functional integration between sensory and attentional large-scale brain networks, while higher rhythms might reflect more local sensory processing. To explore this hypothesis, we used a beamformer technique to reconstruct the neural origin of delta and gamma signals.

#### Effects of cold stimulation at the source level.

**Fig. 5.**
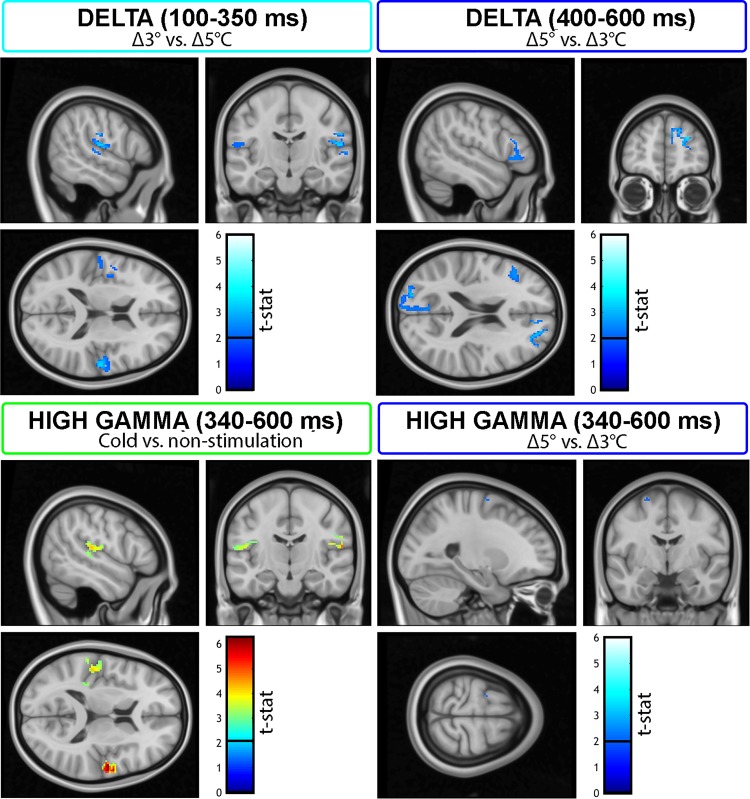
Cortical generators associated with the low- and high-frequency oscillatory effects observed at the scalp level. The neural origin of the early delta oscillatory activity (100–350 ms) was estimated in bilateral operculo-insular regions, with Δ3 vs. Δ5°C associated with larger activations in right (contralateral) operculo-insular sites. Furthermore, the source of the late delta oscillatory activity (400–600 ms) was identified in several brain regions, where Δ5 vs. Δ3°C was associated with greater source strengths in prefrontal, as well as parietooccipital and temporal sites. Finally, the neural origin of cold-related gamma band oscillations between 340 and 600 ms was estimated in bilateral operculo-insular regions. The largest cold vs. nonstimulation effect was observed in right (contralateral) operculo-insular areas. Within the same time window, the difference between the two cooling stimuli was identified in a left motor region. Images were inspected at a threshold of *t* > 2 and plotted on subjects-average T1 image. Color bars indicate *t*-statistic (*t*-stat) at each voxel.

Cortical generators of oscillatory cold-evoked responses were identified for the delta and gamma time-frequency effects observed at the scalp level. In the time window between 100 and 350 ms, delta oscillatory activity during cold vs. nonstimulation was associated with a distributed network of sources, including operculo-insular, parietal, and frontal regions. Specifically, the contrast Δ3 vs. Δ5°C revealed that the main difference in the early delta activity depended on an increased activity in bilateral parietal operculum regions (i.e., second somatosensory cortex; Fig. 5). The strongest cluster was observed in right (contralateral) OP1 and OP4 regions (MNI coordinates: [54 −17 17]). Other smaller clusters were observed in left (ipsilateral) OP4 (MNI coordinates: [−56 −16 14]) and left inferior frontal gyrus (MNI peak coordinates: [−46 38 −7]). The early delta-evoked activity might be associated with cold-related sensory processing in operculum regions.

In the time window between 400 and 600 ms, the neural origin of cold vs. nonstimulation delta band oscillations was also estimated to originate from parietal, frontal, and temporal brain regions. However, the contrast between Δ5 and Δ3°C revealed increased source strength in prefrontal areas, specifically in right superior frontal gyrus and left inferior frontal gyrus (MNI coordinates: [21 44 30] and [−44 24 11]; Fig. 5). This late delta activity might be associated with perceptual or more cognitive processing, and might reflect perceptual and attentional factors related to the processing of stronger cooling signals.

Finally, in the time window between 340 and 600 ms, the neural origin of gamma oscillatory activity during cold vs. nonstimulation was estimated in left and right operculo-insular regions (Fig. 5). Specifically, the regions corresponded to OP1 and OP4 [MNI coordinates: [−50 −27 16] and [55 −11 15]). These coordinates are similar to the ones observed for early delta band activity. The difference between Δ5 and Δ3°C revealed increased source strength in a left motor region (MNI coordinates: [−22 −5 66]; Fig. 5), possibly related to increased motor preparation, when detecting stronger cold signals.

## DISCUSSION

We investigated evoked and oscillatory neural activity associated with cold-evoked responses using magnetoencephalography (MEG) and EEG. Specifically, we characterized the time courses (i.e., evoked fields and potentials) and oscillatory neuronal synchrony associated with innocuous cold thermosensation in humans. We showed cold-evoked responses peaking at around 240 and 500 ms, at similar peak latencies in both MEG and EEG. However, MEG fields but not EEG potentials were differentially modulated by cooling intensity, a result that we link to the increased MEG spatial accuracy in distinguishing between neighboring sources close to the scalp surface (Hämäläinen et al. 1993). The latency of contact cold and heat evoked responses are highly similar (e.g., Madsen et al. 2012) but are of consistently greater duration with respect to laser-evoked potentials [for a review, see Garcia-Larrea et al. (2003)]. These differences are most likely related to stimulus duration, which is of the order of hundreds of milliseconds in the case of contact thermal stimulation but tens of milliseconds in the case of laser stimuli. Innocuous cold stimuli primarily activate A-delta fibers but may also activate C2 fibers, while lower cold temperatures (below 20°C) activate multimodal high-threshold neurons (Ma 2010; McKemy 2013). However, the transmission delays of cold-evoked potentials and fields observed here were consistent with the activation of A-delta fibers (Hüllemann et al. 2016). Furthermore, we did not observe ultraslow EEG or MEG responses compatible with C fiber activity (i.e., latency > 1 s).

A key finding of this study is the identification of widespread changes in oscillatory power in response to cold stimulation, ranging from delta-to-theta synchronization (1–8 Hz), to alpha-to-beta desynchronization (8–30 Hz), to gamma synchronization (30–45 and 55–90 Hz). Previous studies have shown that delta-theta synchronization and alpha desynchronization are also evoked by tonic cold pain (Chang et al. 2002). In general, event-related delta and theta oscillations have been shown to serve active sensory and cognitive functional roles across different sensory domains (Arnal and Giraud 2012; Knyazev 2012). More specifically, delta oscillations are thought to mediate synchronization between the central and autonomic nervous systems and also facilitate the detection of motivationally salient events (Knyazev 2012). Furthermore, the continuum between delta and theta frequencies is implicated in sensory and attentional selection in primary cortical areas (Arnal and Giraud 2012; Schroeder and Lakatos 2009). In the present study, the delta synchronization peaking at 4 Hz likely reflects the integration of ascending signals communicating changes in cold sensory inputs with attentional signals from a distributed network of brain regions. Indeed, we observed that these oscillations were associated not only with cold-related sensory regions (i.e., operculo-insular regions) in an early time window (100–350 ms) but also with frontal regions (i.e., left inferior frontal and right superior frontal areas) at a later processing stage (400–600 ms). This latter effect might reflect the combination of continuous top-down attention required to perform the detection task and stimulus-driven bottom-up attention due to randomized and long interstimulus intervals between consecutive cooling stimuli. Furthermore, cold-related activity was associated with increased gamma synchrony peaking at ~70 Hz (55–90 Hz). High-frequency oscillations are thought to emerge from the coordinated interactions between excitatory and inhibitory neurons in local circuits (Buzsáki and Draguhn 2004) and are commonly observed in primary sensory areas. Here, we identified the neural origin of cold-related gamma synchrony in bilateral operculo-insular regions. This effect suggests a key role of gamma oscillations in mediating local sensory and attentional processing of cold-related signals.

Overall, the topography and neural generators of the cold-related oscillatory activity are congruent with previous electrophysiological and neuroimaging studies. Indeed, cold-evoked potentials and fields are consistently observed over bilateral frontotemporal channels (Chatt and Kenshalo 1979; Jamal et al. 1989; Maihöfner et al. 2002). In one previous MEG study, the timing and topographies of evoked responses was previously linked to contralateral activation of posterior insular cortex, followed by an ipsilateral activation of the same region (Maihöfner et al. 2002). Importantly, with respect to this previous study, our increased sensitivity in detecting multiple sources, beyond operculo-insular regions, can be related to the extended coverage of our MEG sensor arrays (306 vs. 37 sensors) and the choice of source reconstruction method (distributed solution vs. dipoles). Overall, our source reconstruction results are consistent with previous functional studies demonstrating a key role of operculo-insular regions in innocuous cold processing but also reveal the contribution of a more extended network of brain regions, including frontoparietal areas [Craig et al. 2000; Hua et al. 2005; see also Oshiro et al. (2007) and Oshiro et al. (2009)]. Crucially, we showed that cold-related neuronal activity in operculo-insular regions is associated with both low- and high-frequency oscillatory rhythms, mediating functional integration at both local and large-scale brain networks.

### Limitations and Future Directions

In the present study, the source analysis results must be interpreted with caution given that the spatial resolution afforded by MEG decreases as a function of distance (i.e., deeper sources can be localized less accurately). Indeed, here we do not emphasize specific cortical subregions (e.g., between posterior insula and second somatosensory cortex), as it is unlikely that MEG can effectively dissociate these different cortical subregions. To better address this issue, future studies might adopt an EEG-functional MRI multi-imaging approach to take advantage of the excellent temporal and spatial resolution offered by the combination of the two techniques and precisely assess the origin of oscillatory activity associated with cold perception. Furthermore, we were unable to fully distinguish sensory and attentional processing, given that participants were instructed to actively attend to all incoming cold stimulation. Future studies may benefit from the experimental manipulation of a larger range of cooling temperatures and attentional effort to further distinguish the neuromagnetic features that are specifically sensitive to cold sensory processing from more attentional, cognitive, and decisional task-related factors. A second limitation is related to the adopted cold temperature range. Currently, using commercially available devices, cold-evoked responses can be investigated only for a narrow cold temperature range (Madsen et al. 2014). This limitation is linked to the inability of these thermodes to allow steep temperature changes below 30°C. Steep ramps are necessary to activate and accelerate the discharge of a sufficiently large number of cold-sensitive fibers, ensuring synchronized cortical activations that are measured as evoked responses (Jamal et al. 1989). So far, most studies have used custom-made devices that can vary with respect to the extent of the thermode contact area and slope of cooling ramps. Furthermore, they used different stimulus parameters with respect to baseline temperature, range of cold temperature change, and stimulus duration and location. These variables greatly influence the latency and magnitude of cortical evoked responses, rendering comparison between studies problematic. The limited experimental and clinical research on innocuous and noxious cold is thus most likely due to current technical challenges. Nevertheless, the neurophysiological assessment of cold-sensitive pathways has crucial experimental and clinical value.

### Clinical Implications

Currently, laser-evoked potentials are the gold standard for the clinical assessment of small-fiber function and damage in patients with peripheral and central neuropathies (Cruccu et al. 2004; Haanpää et al. 2011). However, laser stimuli activate only a subset of thermosensory and nociceptive small fibers that can contribute to pain symptoms and are subject to dysfunction in the case of small-fiber damage. As a consequence, a thorough examination of small-fiber function and neuropathy should also include the neurophysiological assessment of nonnociceptive cold-sensitive pathways (Hüllemann et al. 2016). Importantly, the testing of cold-sensitive pathways would implicate limited discomfort for the patients, as mild cooling of the skin is usually not associated with pain sensations (Madsen et al. 2014), and may still provide information of important clinical value. Future studies should investigate whether the diagnosis of small-fiber damage in neuropathic pain patients can be supported not only by reduced amplitudes and increased latencies of laser-evoked potentials but also by reduced amplitudes and increased latencies of the cold-evoked potentials identified here.

### Conclusions

In the present study, we identified key EEG and MEG features reflecting the functionality of A-delta nonnociceptive pathways, probed by transient innocuous cold stimuli. Cold-evoked responses peaked at around 240 and 500 ms and resembled the well-known pain-related N1 and P2 potentials in terms of morphology and topographical scalp distribution. Cold-related neuromagnetic activity was characterized by increased synchronization in delta and gamma band oscillations. Intriguingly, the increased synchronization at lower frequency was related to cooling intensity and suggested a role of delta band activity in coordinating between a distributed network of sensory (i.e., operculo-insular) and attentional (i.e., prefrontal) brain regions. In contrast, the increased synchronization at higher frequency appeared to reflect sensory cold processing mediated by parietal operculum regions. These findings have interesting implications for the understanding of the neural processing associated with cold perception in healthy humans and open new venues to identify the neural mechanisms underlying altered cold perception in clinical populations suffering from cold allodynia.

## GRANTS

The authors acknowledge the support of the Danish Council for Independent Research (Grant 6110-00643B; F. Fardo), Novo Nordisk Foundation (Grant NNF14OC0011633; T. S. Jensen and N. B. Finnerup), and the Wellcome Trust (Grant 100227; M. Allen).

## DISCLOSURES

No conflicts of interest, financial or otherwise, are declared by the authors.

## AUTHOR CONTRIBUTIONS

F.F. and N.B.F. conceived and designed research; F.F. and M.C.V. performed experiments; F.F. analyzed data; F.F. and M.A. interpreted results of experiments; F.F. prepared figures; F.F. drafted manuscript; F.F., M.C.V., M.A., T.S.J., and N.B.F. approved final version of manuscript; M.C.V., M.A., T.S.J., and N.B.F. edited and revised manuscript.
